# Sequencing and Staphylococci Identification

**DOI:** 10.3201/eid1202.050962

**Published:** 2006-02

**Authors:** Alexander Mellmann, Karsten Becker, Christof von Eiff, Ursula Keckevoet, Peter Schumann, Dag Harmsen

**Affiliations:** * University Hospital Münster, Münster, Germany;; † Deutsche Sammlung von Mikroorganismen und Zellkulturen, Braunschweig, Germany

**Keywords:** rpoB gene, 16S rDNA, Staphylococcus, small-colony variants, molecular identification, dispatch

## Abstract

The emerging clinical importance of staphylococcal infections prompted us to establish a reference database for partial RNA polymerase B (*rpoB*; nucleotides 1444–1928) gene sequences from type strains of all staphylococcal species and subspecies. This database correctly identified 55 clinical staphylococcal isolates; all were correctly identified at the species level. At the subspecies level, *rpoB* misidentified only 2 isolates.

The emerging clinical importance of *Staphylococcus aureus* and coagulase-negative staphylococci (*1*) in connection with the expanding number of staphylococcal subspecies described requires accurate identification to the subspecies level. Currently, the genus *Staphylococcus* is divided into 36 species and 21 subspecies. Staphylococcal subspecies not included in the databases of commercial identification systems, as well as phenotypic variants (e.g., small-colony variants), are often misidentified (*2*).

We recently described the usefulness of genotypic identification of staphylococcal subspecies by using partial 16S rDNA sequences in comparison with phenotypic tests (*3*). However, the partial 16S rDNA sequences used were not discriminative enough to differentiate all staphylococcal subspecies. When searching for a molecular target for discrimination of staphylococci, several genes have been evaluated, e.g., heat shock protein 60 (*hsp60*) (*4*), superoxide dismutase A (*sodA*) (*5*), and RNA polymerase B (*rpoB*) (*6*). However, these studies concentrated only on a limited number of staphylococcal species. Therefore, a complete reference database of partial *rpoB* gene sequences from type strains (n = 47) and other culture collection strains, including all validly described staphylococcal subspecies, was created for this study. This reference database was then evaluated with clinical isolates. Results were compared with those previously obtained by 16S rDNA sequencing and conventional phenotypic tests.

## The Study

We analyzed 82 type and other culture collection strains encompassing all validly described staphylococcal species (n = 38) and subspecies (n = 21; according to the current List of Bacterial Names with Standing in Nomenclature, updated May 14, 2005) (*7*). Two strains of the recently proposed candidate species *S. pettenkoferi* (*8*) were added to complete the *rpoB* sequence reference database. Using this database, we analyzed 55 clinical staphylococcal isolates collected from human (n = 52) and animal (*S. intermedius*, n = 2; *S. felis*, n = 1) specimens; 6 of the human isolates exhibited the small-colony variant (SCV) phenotype.

This strain collection was previously analyzed by the API ID 32 Staph and VITEK 2 systems (both obtained from bioMérieux, Marcy l'Etoile, France), partial 16S rDNA sequencing, chemotaxonomy, and riboprinting to determine species designation (*3*). The thermal cycling condition to amplify the partial *rpoB* gene (899 bp) was 35 cycles of denaturation at 94°C for 45 s (300 s for the first cycle), annealing (60 s at 52°C), and extension (90 s at 72°C, 600 s for the last cycle). The *Staphylococcus*-specific primers used for amplification and sequencing of *rpoB* are shown in Table 1. Sequencing reactions were performed in a total volume of 10 μL containing 0.5 μL premix (ABI Prism BigDye Terminator v3.0 Ready Reaction Cycle Sequencing Kit, Applied Biosystems, Darmstadt, Germany), 1.8 μL 400 mmol/L Tris-HCl, 10 mmol/L MgCl_2_, 10 pmol sequencing primer, and 2 μL polymerase chain reaction product. The sequencing products were purified by using the Centri-Sep Spin Columns (Princeton Separations, Adelphia, NJ, USA) and analyzed with the ABI Prism 3100 Avant Genetic Analyzer (Applied Biosystems) according to the manufacturer's instructions. For further analysis, nucleotides 1444–1928 (corresponding to *S. aureus rpoB* gene positions of the GenBank accession no. X64172) of the *rpoB* gene were used. The sequences were analyzed by using Ridom TraceEditPro version 1.0 software (Ridom GmbH, Würzburg, Germany). Staphylococcal partial *rpoB* reference sequences determined in this study were deposited in GenBank under accession nos. DQ120729–DQ120752.

**Table 1:** Primers used for amplification and partial sequencing of the partial staphylococcal RNA polymerase B (*rpoB*) gene

Primer	Application	Primer sequence (5´→3´)	Annealing temperature (°C)	Reference
Staph rpoB 1418f*	Amplification and sequencing	CAA TTC ATG GAC CAA GC	52	Modified from 7
Staph rpoB 3554r	Amplification	CCG TCC CAA GTC ATG AAA C	52	*7*
Staph rpoB 1975r*	Sequencing	GCI ACI TGI TCC ATA CCT GT	52	Modified from 7
Staph rpoB 1876r*†	Sequencing	GAG TCA TCI TTY TCT AAG AAT GG	52	This study

*Primers are numbered from the 3´ end of the primer on the forward strand of *Staphylococcus aureus* (GenBank accession no. X64172). †Primer was used for sequencing when primer Staph rpoB 1975r did not work.

**Figure A1:**
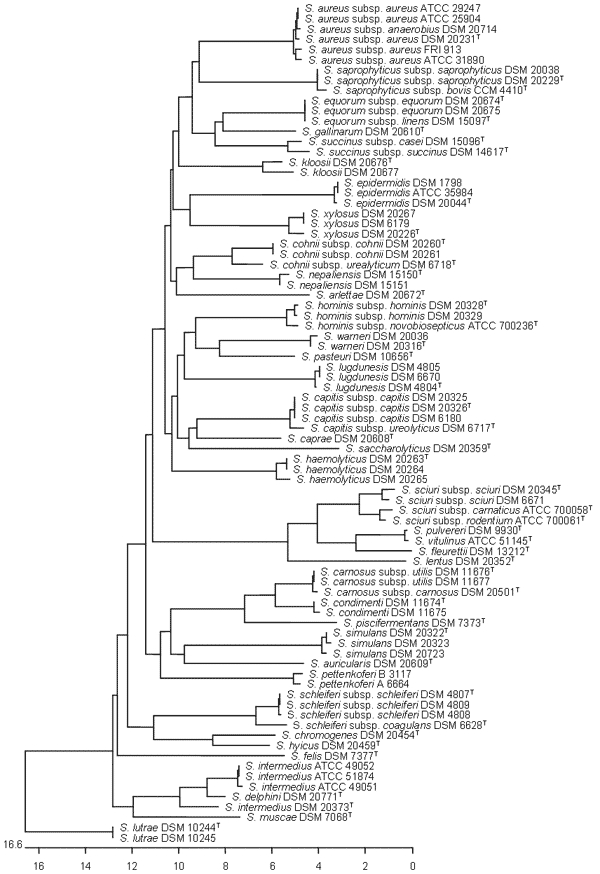
Unrooted neighbor-joining tree based on partial RNA polymerase B (*rpoB*) gene sequences showing the phylogenetic relationship among all validly described species and subspecies of the genus *Staphylococcus* and further staphylococcal culture collection strains (n = 82). The scale bar indicates the evolutionary distance between sequences determined by measuring the lengths of the horizontal lines connecting 2 organisms. ATCC, American Type Culture Collection, Manassas, VA; DSM, Deutsche Sammlung von Mikroorganismen und Zellkulturen, Braunschweig, Germany; T, type strain; FRI, Food Research Institute, University of Wisconsin, Madison, WI, USA; CCM, Česká Sbírka Mikroorganismů, Brno, Czech Republic.

Partial *rpoB* sequences were determined for 82 culture collection strains and 55 clinical isolates. All staphylococcal type strains were distinguishable by *rpoB*; the only exception was the *S. equorum* subspecies that shared the same sequence (Figure A1). The mean pairwise distance of all type and other culture collection strains exhibiting a unique *rpoB* sequence (n = 68) was 13.7% (range 0%–21.4%) and the standard deviation was 3.3%. When assuming a normal distribution for the distances and choosing a reporting criterion >94.0%, the similarity for a distinct species correlates with a statistical error probability of 1.0% (*9*).

The definitive identification of 55 clinical isolates and the *rpoB* gene sequence similarity search results are shown in Table 2. At the species level, the correct species designation for all 55 clinical isolates was made by *rpoB* sequence similarity search (sequence similarity >94.0%). Of 21 clinical isolates belonging to species currently divided into subspecies, 17 isolates were correctly identified to the subspecies level. Subspecies identification for isolates M26 and M53 was unsuccessful by *rpoB* or partial 16S rDNA sequencing, riboprinting, and chemotaxonomy (data not shown). Only isolates M20 and M39 were misidentified by *rpoB* sequencing as *S. saprophyticus* subsp. *saprophyticus* instead of subsp. *bovis*.

**Table 2:** Identification of 55 clinical staphylococcal isolates by using RNA polymerase B (*rpoB*) gene sequencing

Strain	*rpoB* gene (% similarity*)	Definitive identification†
M01	*Staphylococcus arlettae* (100.0)	*S. arlettae*
M02	*S. aureus* subsp. *aureus* (100.0)	*S. aureus* subsp. *aureus*
M03	*S. aureus* subsp. *aureus* (100.0)	*S. aureus* subsp. *aureus*
M04	*S. aureus* subsp. *aureus* (99.8)	*S. aureus* subsp. *aureus*
M05‡	*S. aureus* subsp. *aureus* (99.8)	*S. aureus* subsp. *aureus*
M06	*S. aureus* subsp. *aureus* (100.0)	*S. aureus* subsp. *aureus*
M07‡	*S. aureus* subsp. *aureus* (100.0)	*S. aureus* subsp. *aureus*
M08	*S. haemolyticus* (94.0)	*S. haemolyticus*
M09	*S. epidermidis* (100.0)	*S. epidermidis*
M10	*S. capitis* subsp. *capitis* (100.0)	*S. capitis* subsp. *capitis*
M11	*S. epidermidis* (100.0)	*S. epidermidis*
M12‡	*S. epidermidis* (100.0)	*S. epidermidis*
M13‡	*S. capitis* subsp. *capitis* (99.8)	*S. capitis* subsp. *capitis*
M14	*S. caprae* (99.8)	*S. caprae*
M15	*S. caprae* (99.8)	*S. caprae*
M16	*S. chromogenes* (100.0)	*S. chromogenes*
M17	*S. cohnii* subsp. *cohnii* (99.8)	*S. cohnii* subsp. *cohnii*
M18	*S. cohnii* subsp. *cohnii* (99.8)	*S. cohnii* subsp. *cohnii*
M20	*S. saprophyticus* subsp. *saprophyticus* (100.0)	*S. saprophyticus* subsp. *bovis*
M21	*S. epidermidis* (99.0)	*S. epidermidis*
M22	*S. epidermidis* (100.0)	*S. epidermidis*
M23‡	*S. epidermidis* (100.0)	*S. epidermidis*
M24	*S. epidermidis* (100.0)	*S. epidermidis*
M25‡	*S. epidermidis* (100.0)	*S. epidermidis*
M26	*S. equorum* subsp. *equorum* (100.0); *S. equorum* subsp. *linens* (100.0)	*S. equorum*; subspecies not known
M27	*S. felis* (99.8)	*S. felis*
M28	*S. haemolyticus* (100.0)	*S. haemolyticus*
M29	*S. haemolyticus* (99.8)	*S. haemolyticus*
M30	*S. epidermidis* (100.0)	*S. epidermidis*
M31	*S. epidermidis* (100.0)	*S. epidermidis*
M32	*S. hyicus* (100.0)	*S. hyicus*
M33	*S. intermedius* (100.0)	*S. intermedius*
M34	*S. intermedius* (100.0)	*S. intermedius*
M35	*S. intermedius* (100.0)	*S. intermedius*
M36	*S. xylosus* (100.0)	*S. xylosus*
M37	*S. lugdunensis* (100.0)	*S. lugdunensis*
M38	*S. lugdunensis* (100.0)	*S. lugdunensis*
M39	*S. saprophyticus* subsp. *saprophyticus* (100.0)	*S. saprophyticus* subsp. *bovis*
M40	*S. aureus* subsp. *aureus* (100.0)	*S. aureus* subsp. *aureus*
M41	*S. schleiferi* subsp. *schleiferi* (100.0)	*S. schleiferi* subsp. *schleiferi*
M42	*S. schleiferi* subsp. *schleiferi* (100.0)	*S. schleiferi* subsp. *schleiferi*
M43	*S. sciuri* subsp. *sciuri* (99.8)	*S. sciuri* subsp. *sciuri*
M44	*S. sciuri* subsp. *sciuri* (99.8)	*S. sciuri* subsp. *sciuri*
M45	*S. sciuri* subsp. *sciuri* (100.0)	*S. sciuri* subsp. *sciuri*
M46	*S. simulans* (100.0)	*S. simulans*
M47	*S. hominis* subsp. *novobiosepticus* (99.6)	*S. hominis* subsp. *novobiosepticus*
M48	*S. felis* (99.8)	*S. felis*
M49	*S. felis* (99.8)	*S. felis*
M50	*S. warneri* (95.9)	*S. warneri*
M51	*S. warneri* (95.3)	*S. warneri*
M52	*S. warneri* (96.0)	*S. warneri*
M53	*S. equorum* subsp. *equorum* (99.8); *S. equorum* subsp. *linens* (99.8)	*S. equorum*; subspecies not known
M54	*S. xylosus* (99.0)	*S. xylosus*
M55	*S. xylosus* (97.1)	*S. xylosus*
M56	*S. xylosus* (98.6)	*S. xylosus*

*Similarity in comparison with the reference database. †By phenotypic and genotypic methods as previously published (*4*). ‡Isolate exhibiting the small colony variant phenotype.

## Conclusions

Our previous study demonstrated the superiority of sequence-based methods over phenotypic approaches using the API ID 32 Staph and VITEK 2 systems (*3*). The advantage of a sequence-based method became most evident when differentiating isolates with the SCV phenotype, in which the API ID 32 Staph and VITEK 2 systems misidentified 2 and 4 isolates, respectively. When both sequence-based approaches used were compared, *rpoB* sequencing was superior to partial 16S rDNA identification. Although the 16S rDNA procedure differentiated 50 (90.9%) of all tested clinical isolates at species level, *rpoB* identified 100%. Therefore, if an unknown organism needs to be identified, 16S rDNA sequencing is the method of choice because of the availability of universal primers (*10*). However, if the genus is already known, the *rpoB* method should be used.

Compared with other published molecular probes, *rpoB* showed the highest discriminatory power, e.g., *hsp60* and *sodA* sequencing did not differentiate subspecies of *S. carnosus*, *S. cohnii*, *S. hominis*, *S. schleiferi*, or *S. succinus* (*4**,**5*). In a previous study, *rpoB* sequence-based identification of *Staphylococcus* species has been reported (*6*). However, a limited number of taxa were included, and the primers used were not appropriate to detect all staphylococcal subspecies.

Sequencing of *rpoB* was also used to identify other bacterial species (*11**,**12*). A higher discrimination with *rpoB* sequencing compared with 16S rDNA sequencing has been demonstrated for the genera *Corynebacterium* (*13*) and *Bacillus* (*14*). DNA sequencing is a rapid alternative to biochemical and other phenotypic procedures for the differentiation of bacterial pathogens because of its decreased costs and increased automation (*15*). Thus, *rpoB* is a useful molecular target for differentiating staphylococcal isolates to the species and subspecies level.
